# Comment on: “UGT2B17 modifies drug response in chronic lymphocytic leukaemia”

**DOI:** 10.1038/s41416-020-1005-5

**Published:** 2020-07-24

**Authors:** Spyros I. Papamichos, Christof Jungbauer

**Affiliations:** Blood Service for Vienna, Lower Austria and Burgenland, Austrian Red Cross, Vienna, Austria

**Keywords:** Chronic lymphocytic leukaemia, Cancer genomics, Gene regulation

We read with great interest the article by Allain et al.^1^ and were intrigued by the UGT2B17-mediated direct drug inactivation or indirect anti-leukaemic response modification of various therapeutic agents in chronic lymphocytic leukaemia (CLL). In human, *UGT2B17* gene expression is detected in hepatic tissues as well as in steroid target tissues.^2^ Nonetheless, shown elegantly in the study by Allain et al.^1^ is that in lymphoid cell models and in CLL patients occurs a lineage-inappropriate *UGT2B17* overexpression, predominantly mediated from the alternative *UGT2B17*_n2 transcript that comprises the additional exon 1c, extending the 5′-untranslated region. Lower *UGT2B17* expression from the alternative *UGT2B17*_n3 and n4 transcripts, comprising the alternative 1b exon, was also reported. Of note, all novel transcripts identified encode a functional UGT2B17 protein.^1^ Since UGT2B17 represents a main conjugating enzyme for fludarabine glucuronidation and consequently *UGT2B17* misexpression could affect primary response to first-line treatment with fludarabine in CLL patients, we fully agree with Allain et al.^1^ that the molecular machinery underlying high *UGT2B17* expression in lymphoid cells warrants further clarification. Herein, we present evidence that both *UGT2B17* exons 1b and 1c were evolutionarily derived from endogenous-retrovirus (ERV) sequences and that *UGT2B17* overexpression in B cells of CLL patients is driven by the in cis aberrant activation of long terminal repeats (LTRs) of the ERV1 family.

ERVs represent heritable provirus insertions into the host genome DNA, remnants of exogenous infectious retroviruses.^3^ The typical genomic structure of a retrovirus consists of a *gag*, *pro*, *pol*, and *env* genes flanked by two LTRs that naturally comprise core transcription regulatory elements and transcription factor (TF) binding sites.^3^ The majority of ERVs in human genome has undergone recombination events between the 5′- and 3′-LTRs, resulting in solitary LTRs.^4^ Interestingly, many solitary LTRs have preserved their ancestral promoter function and, when positioned in the sense orientation of an adjacent host gene, could drive ectopic transcription initiation.^4^ Accordingly, because epigenetic silencing represents the predominant path to ERV transcription inactivation, alterations of the epigenetic landscape occurring during cellular transformation events could likely lead to ERV derepression with a significant impact on host gene transcriptional networks, a process designated as onco-exaptation.^5^

Interestingly, molecular evolutionary analysis^6^ of the genomic segment comprising *UGT2B17* exon 1c and its non-canonical proximal promoter sequence reveals that these genetic elements are comprised within a Harlequin-int LTR/ERV1 sequence, of sense orientation to *UGT2B17* gene (Fig. 1). Accordingly, *UGT2B17* exon 1b and the corresponding proximal promoter were also evolutionary derived from an HERVH48-int LTR/ERV1 sequence. However, because the HERVH48-int LTR is of anti-sense orientation (Fig. 1), a less potent promoter activity compared to Harlequin-int LTR would be expected,^4^ as is the case in the study by Allain et al.^1^

**Fig. 1:**
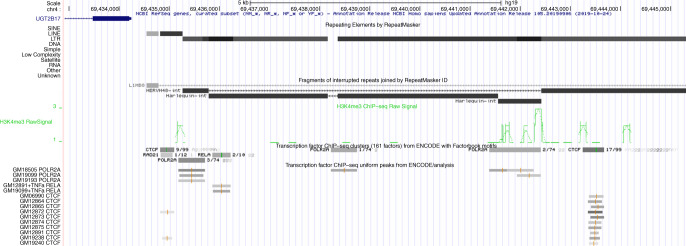
ERV onco-exaptation drives *UGT2B17* ectopic transcription. A screenshot from the UCSC Genome Browser Database (https://genome.ucsc.edu/); shown in the top left corner is the canonical *UGT2B17* exon 1. Upper part: RepeatMasker software analysis^6^ reveals that the genomic segment upstream of *UGT2B17* locus comprises a large cluster of transposable elements. Histone H3 lysine 4 trimethylation (H3K4me3) ChIP-seq data, that is, data for a histone modification highly enriched at active promoters,^7^ were added for the purposes of the study indicating the location of *UGT2B17*_n2 (ChIP-seq peak cluster) and *UGT2B17*_n3/ *UGT2B17*_n4 transcriptional start sites, at −7466 and −1290, respectively (*UGT2B17* translational start codon = +1). Proximal promoters of the transcripts as well as the corresponding exons 1c and 1b are comprised within Harlequin-int LTR and HERVH48-int LTR retroviral sequences, respectively. Lower part: Depicted are TF ChIP-seq data in 91 cell types, generated by the ENCODE project.^7^ Grey boxes delimit the genomic sites occupied by TFs in the ChIP-seq experiments; darkness of the boxes is proportional to the level of enrichment at the site. An orange vertical line within a box reveals the point source of the peak, a green vertical bar denotes the presence of a DNA binding motif from ENCODE Factorbook repository. The symbol(s) and quota appearing next to each box define the cell line(s) where the given TF was found to interact with the genomic sequence. The POLR2A bindings sites, referred to in the text, co-localise with the H3K4me3 ChIP-seq peak clusters and are functional specifically in B-lineage cells; “g” represents a common ENCODE abbreviation for several lymphoid cell lines.^7^ ENCODE computational pipeline identifies also high-scoring binding motifs for RELA and CTCF in the retroelement-shaped genomic segment.

TF chromatin immunoprecipitation and sequencing (ChIP-seq) data in 91 cell types from the ENCODE Project^7^ reveal that the Harlequin-int LTR sequence comprises a binding site for RNA polymerase II subunit A (POLR2A), which, among 74 cell types examined, is functional specifically in the lymphoid cell lines (Fig. 1). Likewise, the HERVH48-int LTR comprises a POLR2A site that is also specific for B cells. Interestingly, short upstream of the HERVH48-int POLR2A site located is a B-lineage functional binding site for RELA proto-oncogene (Fig. 1). In this sense, the identification in this retroelement-derived genomic segment of DNA binding sites for the CCCTC-binding factor (CTCF) is also of importance (Fig. 1). CTCF has the potential to act as transcriptional insulator disabling the interaction between enhancers and non-canonical promoters;^8^ therefore, in a tantalising scenario, a B-lineage-specific aberration of CTCF function could allow the RELA binding site to serve as an unrestricted enhancer of *UGT2B17* inordinate transcription in B-cancerous cells.^9^ These findings are likely in accordance with the results from the study by Allain et al.,^1^ where a co-expression signature of *UGT2B17* with several nuclear factor-κB (NF-κB)-regulated genes was documented, subsequently pointing to NF-κB as a key regulatory “hub point” that targets the non-canonical promoter and drives *UGT2B17* misexpression in CLL cells.

It has been shown that the positive selection of novel hypomethylation motifs, which could subsequently allow the epigenetic derepression of ERV transcription, entails co-evolution of genetic sub-clonal complexity in CLL.^10^ Subsequently, it would be tempting to deduce that the efficacy of *UGT2B17* expression as a prognostic marker of high-risk CLL^2^ is mechanistically linked to the retroviral origin of its ectopic promoters. Most importantly, since the LTR-driven misexpression of *UGT2B17* may impact the effectiveness of fludarabine-based interventions, quantitative PCR-based assays of LTR-driven *UGT2B17* ectopic transcription in cancerous B cells would help tailor fludarabine dosage to better stratified CLL patients.

## Data Availability

The ChIP-seq data reported in this study represent publicly available material, generated from the Encyclopaedia of DNA Elements (ENCODE) Consortium, and are archived at the UCSC Genome Browser Database (https://genome.ucsc.edu/).
